# The Buncefield Oil Depot Fire of 2005: Potential Air-Pollution Health Impacts Under Alternative Meteorological Scenarios

**DOI:** 10.1371/currents.RRN1300

**Published:** 2012-03-12

**Authors:** Richard Mohan, Heather A. Walton, David Thomson, Helen Webster, Paul Wilkinson, Chris Grundy, Virginia Murray, Giovanni Leonardi

**Affiliations:** ^*^Environmental Scientist, Chemical Hazards and Poisons Division (London), Health Protection Agency, UK; ^†^Senior Research Fellow, Science Policy Group, MRC-HPA Centre for Environment and Health, King's College London; ^‡^Research Scientist, Met Office, UK; ^§^Atmospheric dispersion research and response scientist, Met Office, UK; ^¶^Reader in Environmental Epidemiology; ^#^Lecturer in GIS, Department of Social & Environmental Health Research, London School of Hygiene & Tropical Medicine; ^**^Head of Extreme Events and Health Protection Centre for Radiation, Chemicals and Environmental Hazards, Health Protection Agency, London UK and ^††^Head of Epidemiology Dept, Centre for Radiation Chemical and Environmental Hazards, HPA, UK; Honorary Senior Lecturer, Dept of Social and Environmental Health Research, Public Health, London School of Hygiene and Tropical Medicine, UK

## Abstract

Objective: To model the possible air pollution-related health impact of the 2005 oil depot fire at Buncefield, near London, UK, under alternative meteorological conditions to those experienced at the time.

Design: Atmospheric dispersion modelling of the smoke plume was conducted under the range of meteorological conditions occurring throughout 2005 assuming constant particle emission rates. Population exposure to particle concentrations (PM10) was calculated by linking the atmospheric dispersion modelling data (2 km resolution) and postcode population data. Health impacts were estimated using time-series-based exposure-response relationships for PM10 available from the epidemiological literature.

Main outcomes: Estimates of pollution-related deaths brought forward, emergency hospital admissions from respiratory problems and emergency hospital admissions from cardiovascular disease.

Findings: The highest four-day population exposure to PM10 for meteorological data from 2005 was predicted to occur between 5 and 8 August 2005, when northerly winds would have carried the plume towards London and surrounding areas of high population density. On these days, we estimated the additional PM10 exposure would have resulted in around 12 extra deaths brought forward, and around 13 additional emergency hospital admissions and a similar additional number of emergency admissions for cardiovascular disease. These numbers are slightly greater than estimated deaths and emergency admissions attributable to regular anthropogenic PM10 concentrations in south east England over the same four day period.

Conclusions: Although the particle pollution-related health impacts of the Buncefield fire could have been higher under different meteorological conditions, it is unlikely that the impacts would be substantially greater than those attributable to regular anthropogenic particle pollution over the similar period.

Keywords: oil depot fire; health impact; epidemiology; air pollution; explosion; atmospheric dispersion modelling; exposure

## Introduction

At 6 am (BST, British Summer Time) on 11 December 2005 an explosion occurred at the Buncefield oil depot, near Hemel Hempstead, around 20 miles north of London, UK. It damaged commercial buildings on the surrounding industrial estate and nearby housing, and led to a major fire which burned for four days.  There was little increase in air pollution at ground level, probably because of the high temperature of the fire and stable meteorological conditions[1] [2]. An important question for health protection, however, is how much greater might the impacts have been had the fire occurred on a different day.  In this paper we report the results of analyses of pollution dispersion and the associated health impacts had the fire occurred under different meteorological conditions to those experienced at the time.   

## Methods     

The aim was to model the dispersion of PM_10_ (particulate matter passing a sampling inlet with a 50%  efficiency cut-off at 10 µm aerodynamic diameter) over each four consecutive day period during 2005 – i.e. to investigate the impacts had the fire started on any other day of 2005 and burned for four days. We focused on PM_10_ as aircraft measurements taken from within the Buncefield plume showed the plume to consist mainly of black carbon (soot)[1]. The dispersion model predictions of ground level 24-hour average concentrations of PM_10_ were then combined with population data to estimate a worst case scenario in terms of exposure.  The estimated exposure was combined with evidence on exposure-response functions derived from epidemiological time series studies to calculate the potential health burdens.

*Atmospheric dispersion modelling*

Atmospheric dispersion modelling was carried out using the Met Office’s NAME model[3] (Numerical Atmospheric-dispersion Modelling Environment), which was also used during the Buncefield incident to predict the transport and spread of the smoke plume[1]. NAME has a wide range of applications including simulating releases of hazardous materials (chemical, biological, radiological and nuclear), modelling the transport of ash clouds from volcanic eruptions, analysing airborne disease spread, air quality forecasting, pollution episode analysis and identifying pollution source locations and strengths. NAME was driven by three-dimensional meteorological input data obtained from the mesoscale version of the Met Office's Numerical Weather Prediction (NWP) model, which, during 2005, had a horizontal resolution of approximately 12 km and a temporal resolution of one hour.   

Based on the evidence of initial reports, we assumed that the Buncefield site contained 105 million litres of fuel.   We also assumed a constant rate of burning (approximately 400 kg s^-1^), which, using values given by Lemieux et al. (2004)[4], yielded an estimate of 56 kg s^-1^ for the rate of PM_10_ emissions and 16.8 GJ s^-1^ for the rate of heat release[1]. The estimated rate of burning of fuel was derived from an assumption that 40% of the 105 million litres at Buncefield was burnt at a constant rate in the first 24 hours.  This is most likely to overestimate emissions since more recent information suggests that there were in fact only around 39 million litres on site, and not all the fuel was burnt, so actual emissions were almost certainly lower than those used in this study. However, this was deemed a reasonable approach given the significant uncertainty surrounding the emissions.  This rate was applied to four consecutive days in the assessment below. In practice, however, the rate would have declined over time due to fire fighting activities or exhaustion of the fuel supply.  Estimates of the emissions and heat generated per volume of fuel were obtained from previous studies of controlled burning of refined fuels and uncontrolled burning of unrefined fuels (Kuwait oil fires), though some caution is needed in applying the evidence of such studies to the uncontrolled burning of refined fuels which occurred at Buncefield as emissions and heat released depend on the efficiency of the burning and the fuel-to-air ratio[4].

The dispersion model predicted 24-hourly mean PM_10_ concentrations on a 2 km x 2 km grid for a region approximately 200 km (north-south) by 140 km (east-west) centred on the Buncefield oil depot.  This corresponds approximately to an area from Southampton in the south-west to Kings Lynn in the north-east.  The air concentrations were generated for the lowest 250 m of the atmosphere, which, in this study, is thought to provide a good representation of ground level concentrations except very near to the source.  Predicted air concentrations are assumed to be equivalent to concentrations of PM_10_ measured using a gravimetric monitor.  These were divided by 1.3 to give a concentration approximately equivalent to Tapered Element Oscillating Microbalance (TEOM) measurements[5] following the approach adopted by the Committee on the Medical Effects of Air Pollutants (COMEAP)  to use TEOM measurements in its calculation of the health effects of air pollution in the UK[6]. 

*Calculation of health effects*

The predicted 24-hour average PM_10_ concentrations from the dispersion model were linked to post-code level population data obtained from the Office of National Statistics (ONS) Census 2001 dataset. This provided an estimate of the additional PM_10_ exposure for each unit post-code for each day in the year over the study area. The unit postcode in the UK relates on average to around 14 households, and the coordinates of the postcode centroid are available to an accuracy of 100 metres or better. From this, we calculated the attributable health impacts by applying epidemiological exposure-response relationships derived from the literature[6].  

We followed the methods used by the Inter-Departmental Group on Costs and Benefits[7], which are based on the time-series-derived concentration-response functions recommended by the UK’s Committee on the Medical Effects of Air Pollutants (COMEAP)[6].  We calculated the additional effects of the predicted exposure on three health outcomes: 

(i) deaths brought forward

(ii) emergency hospital admissions for respiratory causes

(iii) emergency hospital admissions for cardiovascular causes.

The excess of cases for each of these event types for each postcode and each day were calculated from the following variables: ß, the relative risk (i.e. the ratio of the risks with and without the 1 µg.m^-3^ increase) for a 1 µg.m^-3^ increase in PM_10_; ∆C, the change in concentration of PM_10_ µg.m^-3^ at that postcode; P, the postcode population exposed to ∆ C and E, the daily probability of the event (death, respiratory hospital admission, cardiovascular hospital admission) occurring per person. For relative risks, ß close to 1.0, this can be approximated by: 

∆ E = (ß-1) ∆CPE

The background rate of events and risk coefficient values are summarized in Table 1.

Table 1.  Event rates and concentration-response relationships used in calculations[6]

**Table:** 

	Baseline rate / 100,000 population *per year*	Baseline rate / 100,000 population *per day* (E x 10^5^)	Increase in risk corresponding to a 1 µg.m^-3^ increase in PM_10 _(ß-1)
Deaths (all causes, excluding external causes)	989.7*	2.7	0.075%
Emergency hospital admissions for respiratory disease	979.7^†^	2.68	0.08%
Emergency hospital admissions for cardiovascular disease	981.4^†^	2.69	0.08%

* based on mortality data for 2001 www.statistics.gov.uk † based on Hospital Episode Statistics for 2003/04 www.hesonline.nhs.uk

 The 2 km x 2 km grid box containing the fuel depot itself was removed from the exposure and health effects calculations since the predicted 0 - 250 m air concentrations at the source are not representative of ground level concentrations: concentrations within the rising plume are high, but those outside it are expected to be comparatively low. 

The excess (attributable) cases were calculated for each postcode and each day of the year and the results summed over each four-day period of the year.  This provided an indication of the distribution of population exposures and corresponding health effects for a four day event under the meteorological conditions of 2005.

## Results

The highest four-day mean of population-weighted exposures occurred for the meteorological situation from 5 to 8 August 2005.  This occurred when a northerly wind transported the plume over a densely populated area (exposing approximately 9.3 million people to the plume). Figure 1 shows the numbers of persons exposed to certain concentrations of PM_10_ during this period and also the numbers of people exposed to certain concentrations of PM_10_ on the 17^th^ of July 2005 which was the single day during the year with the highest exposure. The dispersion modelling predicted some extremely high concentrations of PM_10_ (>500 µg.m^-3^), but only in relatively limited areas close to the source of the fire (within ~3 km).  The weather during the period 5 to 8 August 2005 was mostly dry, with average temperatures for the time of year.  This period was initially cloudy with some light rain, becoming sunny later, with fairly light winds from predominantly a north-westerly to northerly direction.

**Figure:**
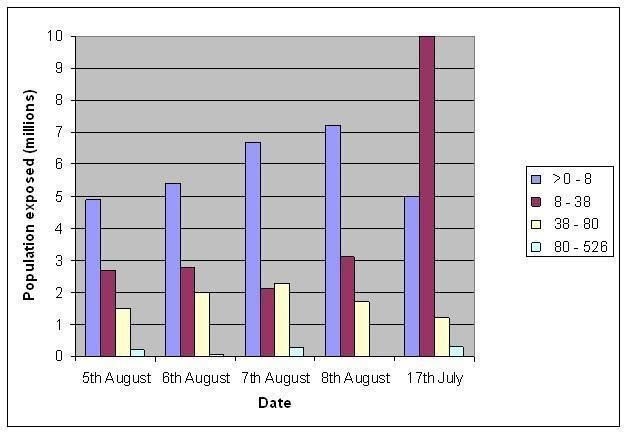
Figure 1. Distribution of estimated ground-level fire-related excess PM_10_ (µg.m^-3^) concentrations for a fire starting on 5 August 2005 (highest 4 day period) and the single day when exposure was highest (17th July 2005)

The attributable health events during this four-day exposure period amounted to around 12 deaths brought forward in time, and a similar number of emergency hospital admissions for respiratory and cardiovascular disease (Table 2).  To put the results in context, we estimated the impact of background anthropogenic PM_10_ concentrations for each day of the month of August 2005 for the area of Eastern, South Eastern and London regions covered by the NAME modelling exercise (see Appendix 1).  The overall number of events attributable to the Buncefield fire was not significantly greater than the deaths and emergency admissions attributable to anthropogenic PM_10_ concentrations over the same time period.

**Table:** 

Table 2. Air pollution-related deaths brought forward and emergency hospital admissions estimated for modelled pollution levels on 5 to 8 August 2005 (corrected to the nearest whole figure)			
Days after onset of fire	Deaths brought forward: all causes	Emergency hospital admissions (additional or brought forward)	
		Admissions for respiratory disease	Admissions for cardiovascular disease
0-1	3	3	3
1-2	3	3	3
2-3	3	4	4
3-4	3	3	3
Total	12	13	13

## Discussion

The results of the analyses we present here provide some context for the events of 11 December 2005. They indicate that a large fire at the Buncefield Oil Depot could have caused greater air pollution-related burdens than were experienced at the time, but that even under unfavourable weather conditions, those burdens would not be substantially greater than those of the ‘usual’ anthropogenic PM_10_ concentrations over a similar period.  

In our models, the highest exposure did not correspond with extreme weather conditions. In calm stable conditions when pollution is able to pool, the heat of the fire enables the plume to rise rapidly through the shallow stable boundary layer into the stable layer above, where it is trapped with little effect on ground level concentrations (the atmospheric boundary layer is the lowest part of the atmosphere which is directly influenced by the ground). With strong wind conditions, there is greater dispersion and relatively low ground-level concentrations. 

Although providing a useful indication of the likely impact of the air pollution exposure from the fire, there are a number of assumptions and uncertainties. There is uncertainty in the emission rates and heat release rates from the fire which are used as input to the dispersion modelling. No adjustments were included to reflect the impact of fire fighting activities on these variables. As a consequence the rate of burning of fuel has probably been overestimated for this modelling. This may not, however, necessarily result in an overestimate of ground level concentrations. If less fuel is burnt then the heat released will be less and the plume may not rise as high, due to buoyancy effects, which may result in higher ground level concentrations.

Changes in predicted PM10 concentrations can simply be scaled in line with changes to the emission release rate if the heat release rate is unchanged. Sensitivity analyses also showed that changes to the heat release rate affect ground level concentrations indirectly; namely, a decrease in the heat release rate will reduce the initial rise of the plume and hence ground level concentrations may increase. NAME simulations in which the heat release rate was varied showed that predicted ground level air concentrations were particularly sensitive to the estimated heat release rate.  

We used only one year of meteorological data which may not necessarily include worst case meteorological conditions. We also assumed outdoor concentrations to be appropriate for calculating exposure, whereas, in reality, the public may stay indoors during such a fire, changing their exposure. Concentration-response functions derived from studies of routine air pollution were assumed to be appropriate for the effects of PM_10_ derived from the oil depot fire. It is relevant to note that our analysis did not consider other health risks that may be important with an oil depot fire, such as direct trauma and injury from explosion.  While the air pollution impacts are likely to be modest, explosion risks may be more important when considering the placement of such depots near areas where people live or work.

In summary, this study has provided an indication of the possible magnitude of the air pollution-related health impacts of a large fire similar to that at the Buncefield oil depot under a range of weather conditions. The results show that, even under less favourable conditions than those experienced at the time of the fire, it is unlikely to have a very major impact on health from exposure to particle pollution.   While this evidence is broadly reassuring for the Buncefield site, we cannot exclude the possibility of appreciably greater air pollution and other health impacts were a similar fire to occur at some of the other oil depots in the UK.   

## Acknowledgments

The authors are grateful to the members of COMEAP for their comments on an earlier version of this work and would also like to thank Harley Quilliam for his help with the data linkage. 

The views represented in this report are those of the authors and not necessarily those of the funding departments.

## Funding information 

This work was funded by the Health Protection Agency, the Met Office and the London School of Hygiene and Tropical Medicine. 

## Competing interests

 The authors have declared that no competing interests exist. 

## Appendix 1

Calculation of impact of background PM_10_ concentrations 

PM_10_ concentrations from air quality monitoring sites in the Eastern, South Eastern and London regions were obtained from the air quality archive [8].   Excluding sites where data were unavailable from 5 to 8 August 2005, the sites used were London Bloomsbury; London Eltham; London Harlington; London Hillingdon; London North Kensington; Norwich; Southend; Thurrock; Canterbury; Harwell; Portsmouth; Reading New Town and Rochester.  Although some of these sites (e.g. Norwich) were not within the area covered by NAME modelling, they were included as being representative of a surrounding area (e.g. East Anglia) that did overlap with the NAME modelling area.  24-hour averages were calculated for each site for each day and then averaged across all sites for that day.  It was then assumed that the entire population in the NAME modelling area (17,517,230 people) was exposed to this ‘all sites average’.

The baseline used for comparison was the concentration of non-anthropogenic PM_10_ (assumed to be about 6 µg.m^-3^) (J Stedman 2008 personal communication). This was subtracted from the ‘all sites average’ concentration to give an estimate of anthropogenic PM_10_. The health impacts were calculated as described in the methodology except that they were not calculated on an individual postcode basis.  The average anthropogenic PM_10_ concentration was used along with the population for the whole area.  This is more approximate than using a population-weighted mean based on mapping concentrations in individual grid squares which was not possible. Figure 2 shows the results using background anthropogenic PM_10_ concentrations during August 2005 for the 3 health outcomes. This shows that the health impacts from the Buncefield fire worst case scenario are similar to those estimated to be caused by background anthropogenic PM_10_.  For further assessment the numbers of deaths brought forward for each day due to background anthropogenic PM_10_ were then subtracted from the monthly average number of deaths brought forward due to background anthropogenic PM_10_  (Figure 3).  This shows that the numbers of health impacts from the Buncefield worst case scenario are within the range of daily fluctuations caused by background PM_10_.   

**Figure:**
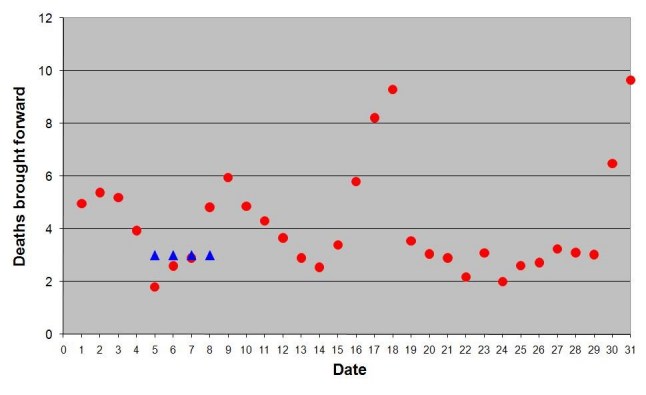
Figure 2. Estimated number of deaths or hospital admissions caused by background anthropogenic air pollution during August 2005 (the blue triangles represent the numbers estimated for the Buncefield worst case scenario for the sake of comparison). (a) Deaths brought forward  

**Figure:**
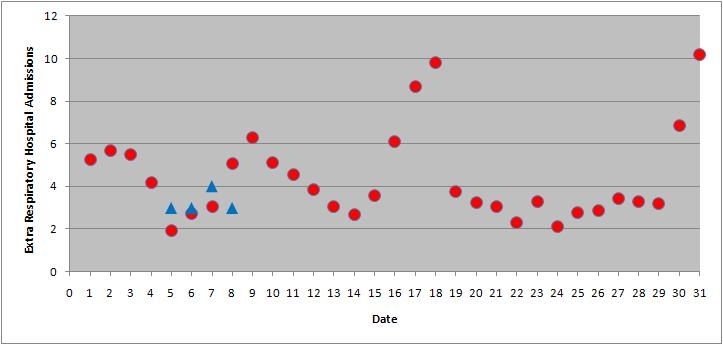
Figure 2. Estimated number of deaths or hospital admissions caused by background anthropogenic air pollution during August 2005 (the blue triangles represent the numbers estimated for the Buncefield worst case scenario for the sake of comparison). (b) Extra respiratory hospital admissions

**Figure:**
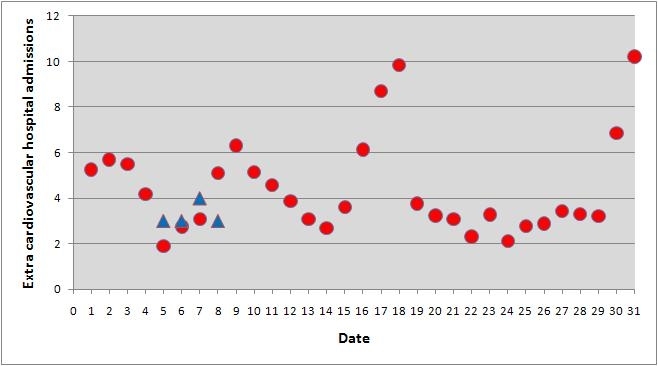
Figure 2. Estimated number of deaths or hospital admissions caused by background anthropogenic air pollution during August 2005 (the blue triangles represent the numbers estimated for the Buncefield worst case scenario for the sake of comparison). (c) Extra cardiovascular hospital admissions

**Figure:**
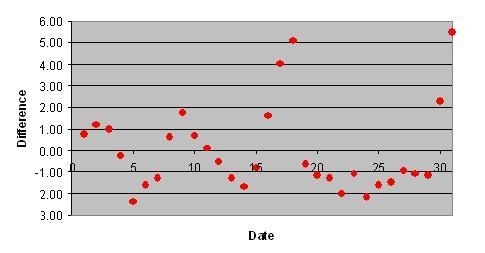
Figure 3. An estimate of the daily fluctuations in the number of deaths brought forward in the south east of England caused by daily changes in background PM_10_ during August 2005 (each dot refers to the difference between the number of deaths brought forward on that day and the average number of deaths brought forward for the month of August 2005)   
